# A Review of Potential Therapies to Attenuate Bone Mineral Density Loss in Obese Individuals Prior to Total Joint Replacement Surgery

**DOI:** 10.7759/cureus.38146

**Published:** 2023-04-26

**Authors:** Charlotte von Hoffmann, Kayla Penta, Amanpreet Singh

**Affiliations:** 1 College of Medicine, Lake Erie College of Osteopathic Medicine, Elmira, USA; 2 Pediatrics, Connecticut Children's Medical Center, Hartford, USA

**Keywords:** calcitonin, bisphosphonate, estrogen, parathyroid hormone, exercise therapy, osteoporosis, total joint replacement surgery, obesity, bone mineral density

## Abstract

The focus of this review is to examine therapeutic interventions which may be used to increase bone mineral density (BMD), reduce bone loss, and ultimately reduce complications in obese patients prior to total joint replacement (TJR). It is recommended that obese patients lose weight prior to surgery to reduce post-surgical complications, but weight loss can also increase bone loss and fracture risk in older individuals. In this review, we investigate potential therapies to improve bone density and reduce bone loss including exercise therapy, parathyroid hormone (PTH), estrogen, bisphosphonate, and calcitonin treatment in obese patients prior to TJR. Our review of existing literature found that treatment with PTH increased total body BMD in both men and women with osteoporosis; exercise therapy in combination with weight loss prevents the weight loss-induced increase in bone turnover and attenuates the weight loss-induced decrease in BMD; and estrogen, bisphosphonate, and calcitonin reduce bone resorption.

## Introduction and background

In recent years, obesity has become a worldwide epidemic; according to the Centers for Disease Control and Prevention, from 1999-2000 through 2017-2020, US obesity prevalence increased from 30.5% to 41.9% [1]. Obesity is known to lead to an increased risk of various adverse health outcomes, such as type 2 diabetes, cardiovascular disease, and certain forms of cancer [2]. Additionally, obesity increases the risk of post-surgical complications, including infection, pulmonary embolism, acute myocardial infarction, deep vein thrombosis, respiratory failure, acute postoperative cerebrovascular accident, postoperative stiffness, postoperative stiffness requiring manipulation under anesthesia, pneumonia, acute renal failure, acute cholecystitis, and the need for postoperative blood transfusion [3]. Both the multitude and severity of complications resulting from obesity highlight the importance of exploring therapies to improve post-surgical outcomes in such patients.

There are multiple factors that can affect an individual’s bone mineral density (BMD). It has been demonstrated that a higher body weight can lead to an increased BMD as a result of either obesity or increased muscle mass due to increased physical activity [2]. Prior studies have shown that a 10 kg increase in body weight is associated with approximately a 1% increase in BMD in both men and women and across cultures [4]. It is thought that the increased mechanical load that heavier individuals exert on weight-bearing bones may provide an explanation as to why some obese patients have higher BMD than their non-obese counterparts [4]. Recent studies, however, have found that obesity may in fact accelerate bone loss. A study of two large population groups concluded that the strengthening effects of weight on bone density were the result of elevated muscle mass and that when the mechanical loading effect of body weight on bone mass is adjusted, increased fat mass is associated with decreased bone mass [5].

Since increased amounts of adipose tissue have been shown to have negative consequences on bone density and the risk of fracture, it is often recommended that obese individuals reduce their weight prior to surgery in order to decrease the risk of adverse consequences [2]. Weight loss, however, can also increase bone loss and fracture risk in older individuals. Certain weight loss methods, such as diet, medications, and exercise, can reduce the bone loss that is associated with weight loss [2]. Thus, it is critical that future weight loss therapies include methods that mitigate the effect of weight loss on BMD and fracture risk.

There are several factors that regulate BMD and bone formation. Normal physiological processes are responsible for maintaining proper bone homeostasis; thus, disruption of these processes by factors such as obesity may lead to adverse effects on bone. Bone formation is balanced with bone resorption, and they are often co-regulated by processes such as calcium homeostasis, adiposity, and hormone signaling [6]. When these processes are misregulated, bone resorption occurs more rapidly than bone formation due to the shorter lifespan of osteoclasts versus active osteoblasts, facilitating a potential decrease in bone density. Moreover, the replacement of osteoblasts and osteoclasts is coordinated at the earlier progenitor level; adipocytes and osteoblasts are derived from the same mesenchymal stem cell [6]. Normally, the differentiation and proliferation of these two cell types are balanced. Obesity increases the differentiation and proliferation of adipocytes while decreasing the differentiation and proliferation of osteoblasts. Thus, the shift away from osteoblast production in obese individuals results in a decrease in bone formation [7].

Another potential mechanism responsible for obesity’s effect on increased bone resorption is mediated by the receptor activator of nuclear factor kappa beta/osteoprotegerin/receptor activator of nuclear factor kappa (RANKL/OPG/RANK) pathway. Decreased bone marrow osteoblastogenesis increases bone marrow adipogenesis, leading to an increase in adipocytes and, consequently, an increase in cytokines affecting the RANKL/OPG/RANK pathway. In the presence of macrophage colony-stimulating factor, RANKL bound to RANK increases osteoclast activity [7]. Other factors, such as an increase in oxidative stress, can also lead to an increased inflammatory response, which in turn increases bone resorption via cytokine-mediated stimulation of the RANKL/OPG/RANK pathway [7].

There are several mechanisms by which obesity may lead to osteoarthritis. Proper joint homeostasis is achieved through a balance between cartilage degradation and regeneration that is primarily regulated by resident chondrocytes [8]. Several factors can disrupt this joint homeostasis such as trauma (single occurrence or chronic micro-traumatic events), dietary intake, and obesity. Results of a recent study showed that subjects with a high BMI had increased tibiofemoral cartilage strain and decreased tibial cartilage thickness versus subjects with a normal BMI [9]. Moreover, increased body fat percentage was found to have a positive correlation with tibiofemoral cartilage strain and a negative correlation with tibial cartilage thickness [9]. If joint homeostasis is dismantled, proteolytic enzymes are no longer balanced by proteolytic inhibitors which leads to a breakdown of the cartilage matrix. In turn, matrix fragments enter the synovial space. As a result, synovial macrophages attempt to phagocytize the fragments, leading to the secretion of local proteases and proinflammatory cytokines. The outcome of these events is a chronic inflammation within the synovial space, culminating in further inflammation and degradation.

As mentioned previously, calcium plays a major role in bone metabolism. Calcium regulates bone metabolism to stimulate bone development. When serum calcium levels are low, parathyroid hormone (PTH) is released from the parathyroid glands and acts at the location of many target organs, specifically the kidneys to increase absorption of calcium, the intestines to increase calcium reabsorption, and the bone to increase bone resorption, all with the purpose of increasing serum calcium levels [10]. A high-fat diet associated with obese patients leads to decreased gastrointestinal calcium absorption, resulting in a decreased availability of calcium for bone formation. The body then compensates by increasing bone resorption in order to increase serum calcium levels [7]. Ultimately, a high-fat diet associated with obesity can lead to reduced levels of calcium, which is a crucial component of bone.

In addition to calcium, certain hormones, such as adiponectin and leptin, are involved in the regulation of bone density [2]. Adiponectin is an adipocyte-derived hormone that regulates insulin sensitivity and energy homeostasis [7]. Furthermore, studies have shown that increased adiponectin may have a protective effect on BMD [11]. Leptin is a hormone that promotes satiety. Patients with increased adiposity display increased serum leptin and decreased serum adiponectin [7]. Increased serum leptin leads to an increase in the transport of macrophages to adipose tissue. Decreased adiponectin promotes adhesion of the macrophages to endothelial cells where they release RANKL/OPG/RANK pathway-stimulating cytokines, specifically TNF-α, IL-1β, and IL-6. This, in turn, leads to the stimulation of inflammatory pathways, which increase bone resorption [6]. Overall, the increase in serum leptin and decrease in serum adiponectin as a result of increased adiposity may lead to increased bone resorption and a reduction in protective effects on bone.

Low BMD prior to surgery does not only affect post-surgical outcomes; it can make it impossible to perform a successful total joint replacement (TJR) if the bone is too brittle to support the insertion of a prosthetic joint [12]. A study found that femoral implant loosening is associated with regional decreases in BMD and that low BMD may predispose patients to implant loosening by providing insufficient implant support [12]. Thus, prophylactic therapies directed toward preventing bone loss may be efficacious in improving surgical outcomes of TJR. The purpose of this review is to explore potential therapeutic approaches to improve BMD in obese patients prior to TJR.

## Review

Methods

This review compiled findings based on the results from numerous studies found via a search through the PubMed database. Key search words included “obesity,” “bone metabolism,” “therapy,” “bone mineral density,” and “total joint replacement.” Using these keywords, 136,793 papers resulted. Further criteria for selection included (1) randomized controlled trials and (2) published after 2000. With this refined inclusion criteria, 6,776 papers resulted. Abstracts were reviewed, and the range of papers selected was then narrowed based on their use of BMD as a primary endpoint. Among those papers that were chosen, many were noted to have detailed the association between obesity, bone metabolism, and BMD, mentioning the roles of exercise, certain medications, and diet in weight fluctuations. Once potential therapies were identified, new key search words were chosen in conjunction with “bone mineral density,” including “exercise,” “parathyroid hormone,” “estrogen,” “bisphosphonate,” and “calcitonin.” Therefore, the final inclusion criteria included the following: (1) randomized controlled trials, (2) published after 2000, (3) primary endpoint of BMD, and (4) keywords. As a result, six papers were reviewed that detailed the effects of those potential therapies on BMD.

Exercise therapy

It has been previously reported that intentional weight loss can lead to the loss of BMD, especially in older adults. Research has shown that incorporating exercise training (ET) with diet-induced weight loss reduces the loss of BMD. Many studies have attempted to discover the most effective method of maintaining BMD during intentional weight loss in older adults [13].

A study by Shah et al. aimed to determine the effects of diet and ET on BMD in obese older adults [13]. Participants were randomized to one of four groups for 52 weeks: (1) a control group that maintained a normal diet and activities, (2) diet group with a balanced diet, resulting in a decrease of about 500-750 kcal per day, (3) exercise group that had 90-minute sessions three days per week, and (4) a combined diet-exercise group with both ET and weight management therapy. All participants were given daily calcium and vitamin D supplements. Dual-energy X-ray absorptiometry was used to measure BMD of the whole body, proximal femur, and lumbar spine at baseline, 6 months, and 12 months [13]. Results showed that body weight decreased by 9.6% in the diet group and by 9.4% in the diet-exercise group, not in the exercise (−1%) and control (−0.2%) groups (between-group p < 0.001). Despite similar weight loss between groups, results showed that there was less bone loss at the hip in the diet-exercise group than in the diet group, while there was an increase in BMD in the exercise group; similar results were observed in BMD of the trochanter and femoral neck. Whole body mineral content, however, was unchanged. This study demonstrated that although diet-induced weight loss decreased BMD, ET increased BMD [13]. The most important finding was that incorporating ET with diet-induced weight loss attenuated BMD loss [13].

A study by Beavers et al. [14] sought to determine the effectiveness of resistance training (RT) versus aerobic training (AT) in maintaining BMD (or attenuating BMD loss) in obese and overweight adults during caloric restriction (CR). This retrospective study compared data from two randomized controlled trials. Participants engaged in either an RT program three days per week on weight-stack machines or an AT program four days per week consisting of walking on a treadmill for 30 minutes. Both groups were provided with a controlled diet as well as calcium and vitamin D supplements. All variables were measured within 3 weeks before the start of the training programs and one week after it ended. Dual-energy X-ray absorptiometry was used to measure BMD and percent total body fat of the lumbar spine (L1-L4) and left hip, including the intertrochanter space, trochanter, and femoral neck. Results showed that BMD of the hip and femoral neck was unaffected in individuals in the RT+CR program, while BMD decreased slightly in individuals in the AT+CR program. During follow-up, the BMD of the lumbar spine was increased in both the RT and AT programs, with no significant difference between them. This study demonstrated that including RT with a calorie-restricted diet may be more effective in attenuating BMD loss in the hip of older adults than AT [14].

In combination, these studies prove to be clinically significant as obese individuals undergoing weight loss prior to TJR can effectively preserve BMD by incorporating exercise, especially RT, into their weight loss agendas.

PTH

PTH has previously been shown to prevent, stop, or partially reverse bone loss in animals and humans [15]. In animals, PTH increases both bone mass and bone strength, suggesting that PTH as therapy may provide protection against fractures in humans. Depending on the route of administration, PTH can either increase or decrease bone mass. Both continuous infusions and daily subcutaneous injections stimulate bone formation. Continuous infusions result in constitutive elevation of serum PTH, while daily injections lead to transient increases in serum PTH [15]. Notably, continuous infusions result in greater bone resorption than daily injections, suggesting that transient as opposed to persistent serum increases in PTH may have a greater protective effect on bone [15].

A study by Neer et al. [15] showed that treatment of postmenopausal osteoporosis with PTH (1-34), also known as teriparatide, decreased the risk of vertebral and nonvertebral fractures, increased vertebral, femoral, and total-body BMD, and is well tolerated by subjects. The study consisted of 1637 postmenopausal women with prior vertebral fractures who were randomly assigned to receive 20 or 40 μg of PTH (1-34) or placebo administered subcutaneously each day. BMD of the lumbar spine, proximal femur, and radius and the total-body BMD was measured using dual-energy X-ray absorptiometry. Bone density of the spine was measured at baseline, 12 months, and 18 months, as well as at the end of the study in all women. Bone density of the hips (in all women), forearms (in a subgroup), and total body (in a subgroup) were measured at baseline, at 12 months, and at the end of the study. Results showed that treatment with PTH (1-34) significantly increased BMD of the spine and hip and in total-body bone mineral in a dose-dependent manner. Daily treatment with PTH (1-34) reduced the risk of nonvertebral fractures by 35% at the 20-μg dose and by 40% at the 40-μg dose and reduced the risk of nonvertebral fragility fractures by 53% and 54%, respectively [15].

A study by Orwoll et al. [16] showed that teriparatide (recombinant human PTH (1-34)) increased BMD in men with osteoporosis. The study consisted of 437 men with spine or hip BMD more than two SD below the young adult male mean who were randomly placed into three groups (daily injections of placebo, teriparatide 20 ug, or teriparatide 40 ug). BMD was assessed using dual-energy X-ray absorptiometry. After three months of teriparatide treatment, spine BMD was greater than in placebo subjects. By 11 months, spine BMD was increased by 5.9% (20 ug) and 9.0% (40 ug) above baseline (p < 0.001 vs. placebo for both comparisons). Femoral neck BMD increased 1.5% (20 ug; p = 0.029) and 2.9% (40 ug; p < 0.001), and whole body bone mineral content increased 0.6% (20 ug; p = 0.021) and 0.9% (40 ug; p = 0.005) above baseline in the teriparatide subjects [16].

Together these findings indicate that treatment with PTH (1-34) increased BMD in both men and women and could provide a potential therapy for increasing BMD in obese patients prior to TJR.

Estrogen, bisphosphonate, and calcitonin

There are many elements that can act as potential therapeutic targets to inhibit bone resorption, specifically estrogen, bisphosphonate, and calcitonin. Estrogen inhibits bone resorption by reducing osteoclast generation. Therapeutically, estrogen has the ability to inhibit both bone loss and bone turnover while showing increases in BMD. There are certain risks associated with the use of estrogen as a therapy because it has known effects on other tissues, leading to potential undesirable side effects. There are limits to the therapeutic uses of estrogen as a treatment for osteoporosis due to the risk for uterine cancer, breast cancer, and potential increased thromboembolic events. No long-term studies have been conducted demonstrating estrogen’s ability to reduce bone fracture risk [6].

Selective estrogen receptor modulators (SERMs) are agents that act as full or partial estrogen agonists with effects on various tissues. One major action is that they inhibit bone resorption by reducing osteoclast generation. Unlike estrogen, SERMs are able to exhibit tissue-selective pharmacology due to their ability to create unique ligand-induced conformational change on estrogen receptors of specific tissues. SERMs bind with high affinity to these estrogen receptors, blocking the production of cytokines that promote osteoclast differentiation. One type of SERM, known as raloxifene, showed a reduction in fracture risk as well as a reduction in new breast cancers. On the other hand, SERMs are not without risk; tamoxifen, for example, showed an increased uterotrophic effect and increased risk of uterine cancer [6]. Though not without risk, SERMs may provide a therapeutic option for reducing bone resorption associated with obesity.

In addition to estrogen, bisphosphonates can inhibit bone resorption by reducing osteoclast activity when used therapeutically. Bisphosphonates inactivate osteoclasts, which then undergo apoptosis, resulting in reduced bone resorption, lower bone turnover, and positive bone balance. Bisphosphonates are presumably the most effective inhibitor of bone resorption because they function as analogs of pyrophosphate (P-O-P) by replacing oxygen with carbon and various side chains. One type of bisphosphonate, known as alendronate, was the first inhibitor that showed a reduction in fractures of both the spine and hip. Similarly, risedronate showed a reduction in the spine and all-site fractures [6].

Calcitonin has also been known to inhibit bone resorption. As mentioned previously, a high-fat diet leads to decreased gastrointestinal calcium absorption, resulting in a decreased availability of calcium for bone formation [7]. Calcitonin acts to fine-tune extracellular calcium regulation, which may be beneficial in obese individuals with these high-fat diets. However, it is important to note that a potential adverse consequence of therapeutic calcitonin use is calcitonin-induced loss of receptors specific to calcitonin, resulting in hormone-induced resistance [6].

In addition to these three hormones, there are other potential targets to increase BMD prior to joint replacement surgery that are under investigation. Statins, which act as inhibitors of hydroxy-methyl-glutaryl-CoA (HMG-CoA) reductase, have been found to enhance bone formation. Statins, such as lovastatin and simvastatin, are widely available as they are currently being used as drugs to lower cholesterol. In animal models, they have been found to increase bone formation and bone volume; the effects on human bone development, however, are still under research [6].

## Conclusions

In this review, we evaluated the effect of obesity on bone metabolism and regulation. We also discussed the therapeutic potential of various treatments for improving BMD in obese patients prior to TJR. Based on our findings, future research should examine the effectiveness of weight loss therapy in combination with hormonal therapy to reduce the occurrence of post-surgical complications due to obesity while simultaneously mitigating the adverse effects of both excess adipose tissue and weight loss on BMD. Our review of the existing literature found that treatment with PTH increased total body BMD in both men and women with osteoporosis; RT in combination with weight loss prevents the weight loss-induced increase in bone turnover and attenuates the weight loss-induced decrease in BMD; and estrogen, bisphosphonate, and calcitonin act to reduce bone resorption to attenuate bone loss.

Because obesity is associated with multiple post-surgical complications, it should be beneficial for patients to lose weight prior to TJR surgery. On the other hand, weight loss may lead to a decrease in BMD, which may complicate or inhibit the ability of the surgery to be performed. The weight loss should specifically be structured in a manner that combines exercise, most notably RT, with CR, but there is still further research needed to determine the most effective weight loss method for maintaining BMD. In addition to a weight loss regimen, daily subcutaneous PTH injections, leading to a transient increase in serum PTH levels, may provide a protective effect on the weight-loss-induced decrease of BMD. Moreover, estrogen, bisphosphonate, and calcitonin may be used for the purpose of a reduction in bone resorption, providing a similar protective measure. Thus, weight loss in combination with hormone therapy aimed at increasing BMD prior to surgery may provide a therapy, which can improve surgical outcomes on multiple levels.
